# Minimally Invasive Tube Surgery (MITS)—A Novel Method in Glaucoma Drainage Device Implantation

**DOI:** 10.3390/jcm13216590

**Published:** 2024-11-01

**Authors:** Bharpoor Singh, Andrew J. Swampillai, Mrudula Utukuri, Eduardo M. Normando, Ahmed Mazrouaa, Ahmed Al-Nahrawy, Faisal Ahmed

**Affiliations:** 1 Western Eye Hospital, 153-173 Marylebone Road, London NW1 5QH, UK; 2 Imperial College Ophthalmology Research Group, Department of Surgery and Cancer, Imperial College, London NW1 5QH, UK; 3 Eye Unit, Princess Alexandra Hospital NHS Trust, Harlow CM20 1QX, UK

**Keywords:** glaucoma drainage device, eyePlate-300, minimally invasive tube surgery, MITS technique, aqueous shunt device

## Abstract

**Background/Objectives**: We introduce a new surgical technique for inserting the eyePlate-300—a glaucoma drainage device (GDD). The flexibility of the eyePlate-300 allows for folding and insertion through a smaller conjunctival incision. The procedure is termed minimally invasive tube surgery (MITS). **Methods**: We performed a retrospective analysis of thirteen eyes to assess the efficacy of the MITS technique in a large London tertiary teaching eye hospital with 12 months follow-up. The primary outcome was complete success: defined as ‘IOP > 5 mmHg, <21 mmHg and off drops’. Secondary outcomes: best corrected visual acuity at 12 months, complications, a reduction in the number of drops and need for further pressure-lowering surgery. **Results**: Twelve eyes (92%) achieved a lower IOP. The mean pre-operative IOP was 35.69 mmHg (20–53 mmHg); post-operatively, it was reduced to 11.08 mmHg (5–20 mmHg) (*p* < 0.001). The mean pre-operative drops were 3.462 (3–4), reducing to 0.85 (0–3) at 12 months (*p* < 0.001). There was no change in the mean BCVA. No intraoperative complications were recorded, and no further IOP lowering surgeries were required. **Conclusions**: The MITS technique facilitates the implantation of a glaucoma drainage device through a smaller, less traumatic incision. Preliminary one-year data suggest that the procedure may be safe and effective.

## 1. Introduction

Glaucoma drainage device (GDD) history dates back to 1906 when Rollett first described using horse hair as a seton to drain aqueous humour into the subconjunctival space [1]. Following this, Molteno, in 1969, created the first drainage device with a plate attached to reduce the risk of post-operative hypotony. In the 1990s, both George Baerveldt and Mateen Ahmed [2,3] released their devices, and iterations of their initial devices are still the most popular used devices. Interestingly, there had been very little innovation into a new tube design until the five-year data from the Tube Versus Trabeculectomy (TVT) study showed tube surgery having higher success rates compared with trabeculectomy with Mitomycin C [4].

More recently, new GDD designs have been released into the market, including the eyePlate series (Rheon Medical, Laussane, Switzerland), Ahmed ClearPath (NewWorld Medical Inc., Rancho Cucamonga, CA, USA), and the Paul^®^ Glaucoma Implant (Advanced Ophthalmic Innovations, Singapore) [5]. These plates provide a fresh alternative to tube surgery and open the field to innovative ways of implantation. In particular, the eyePlate series and Ahmed ClearPath have flexible plates to facilitate a minimally invasive insertion onto the sclera.

Traditionally, a GDD such as the Ahmed FP-7 or Baerveldt BG 101–350 (Johnson and Johnson, Santa Ana, CA, USA) is placed in the superotemporal quadrant. A large limbal peritomy is created, extending from the superior rectus to the lateral rectus. This is typically followed by two relieving conjunctival radial relieving incisions to improve exposure. When implanting the Baerveldt 350 plate, the lateral and superior recti muscles need to be isolated and are often tied to ensure the placement of the plate underneath the extraocular muscles.

We describe a novel method of the minimally invasive tube surgery (MITS) technique for implanting a GDD with the use of the eyePlate-300 from Rheon Medical [6]. Our novel method vastly reduces the need for a large limbal conjunctival peritomy, preserving corneal limbal anatomy, reducing tissue trauma, and potentially reducing surgical time.

### New Technique

The senior surgeon, Faisal Ahmed, has devised an innovative method termed minimally invasive tube surgery (MITS), amidst the constraints imposed by the COVID-19 lockdown.

**Figure 1:**
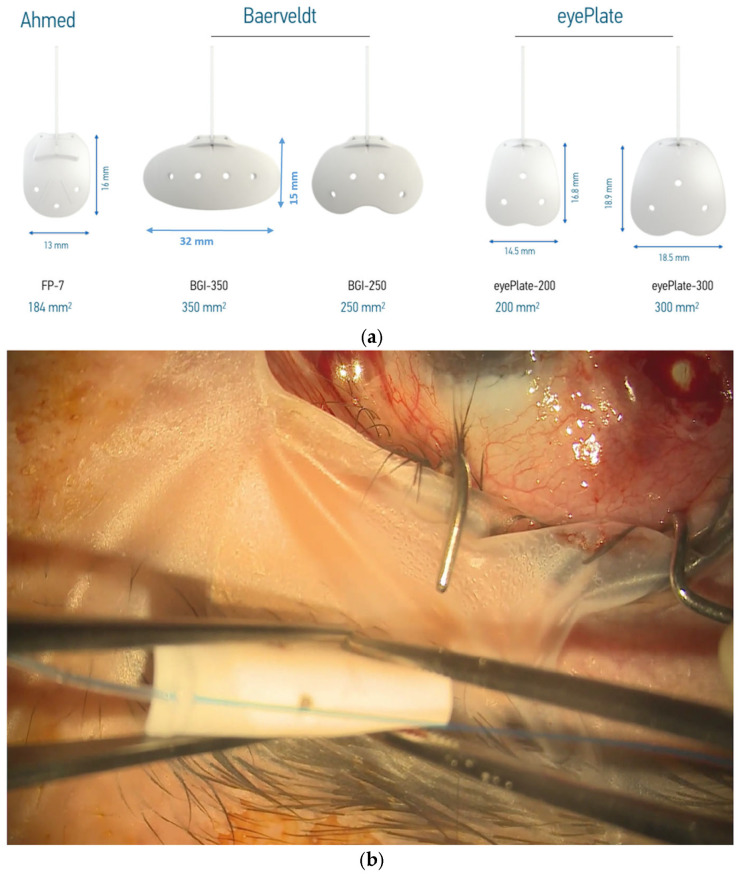
(**a**) eyePlate series compared to the Baerveldt and Ahmed valve implants. Note the rectangular structure of the eyePlate, which can be inserted between the recti muscles. (**b**) eyePlate-300 is flexible and able to be folded. Here, you can see it being folded “Taco Style” before being inserted into the radial pocket.

The eyePlate (Figure 1a) is available in two sizes; in this case series, the larger eyePlate-300 is exclusively used. The eyePlate is non-valved, with the tube and plate being made from silicone.

Despite the comparatively narrow width of the eyePlate-300 at 18.5 mm, it still maintains a large plate surface area due to the larger vertical portion. This is important as the plate does not need to be placed under the recti muscles, unlike the Baerveldt 350. In addition, the eyePlate-300 end plate is thinner (0.8 mm) compared to the Baerveldt (0.9 mm) and Ahmed FP7 plate (1.0 mm). As aforementioned, the eyePlate-300 endplate is more flexible compared to the much more rigid Ahmed FP7 and the Baerveldt 350, which allows it to be rolled ‘Taco Style’ in order to facilitate minimally invasive surgery.

The eyePlate-300 is ideally placed in the superotemporal quadrant. A 7/0 silk corneal traction suture is placed, and the eye is retracted inferonasally to expose the superotemporal quadrant. To create the conjunctival pocket, a subconjunctival injection of bupivacaine with adrenaline is injected behind the limbus mid-way between the superior and lateral rectus. This step helps to ensure adequate exposure of the conjunctiva and induces haemostasis. A vertical radial incision is then made 5 mm behind the corneal limbus with spring scissors extending posteriorly, approximately 8–10 mm in length. The conjunctiva is then dissected through this pocket to expose the bare sclera up to the lateral and superior recti. Haemostasis is further augmented with the use of cautery as required.

**Figure 2:**
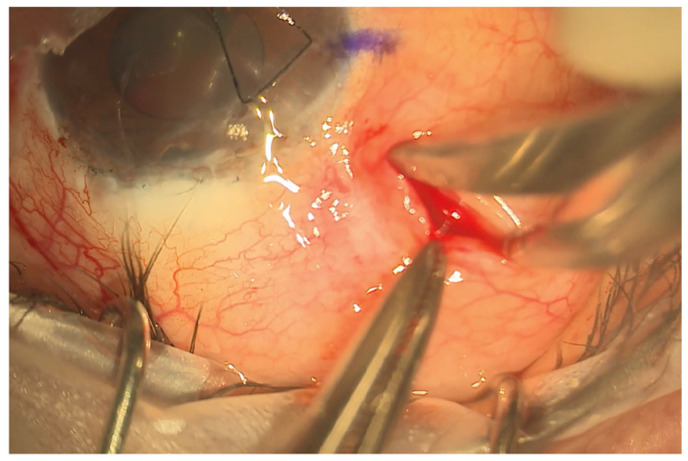
Limbal sparing radial pocket. This is placed 5 mm behind the limbus and extended approximately 8–10 mm posteriorly.

A 4/0 prolene suture is then fed into the tube to stent the eyePlate-300 tube. The eyePlate-300 can then be manipulated with Moorfields and Jayles forceps to be folded “Taco Style’ (Figure 1b) before being inserted on the sclera through the small conjunctival pocket (Figure 2). Care should be taken to unfold the plate under the tenons. Identification of the lateral and superior rectus muscles should take place to ensure the plate sits between the muscles, therefore minimising the risk of post-operative diplopia.

The eyePlate-300 is then sutured via the two eyelets on the plate to the sclera with a 9/0 prolene suture. After trimming the tube to a bevel, the tube is inserted into the anterior chamber via a sclerostomy, which is made 2 mm from the limbus with a 25-gauge needle. The tube is then sutured to the sclera with a 9/0 prolene box suture. An occluding 7/0 vicryl suture is tied around the tube to help prevent early hypotony.

**Figure 3:**
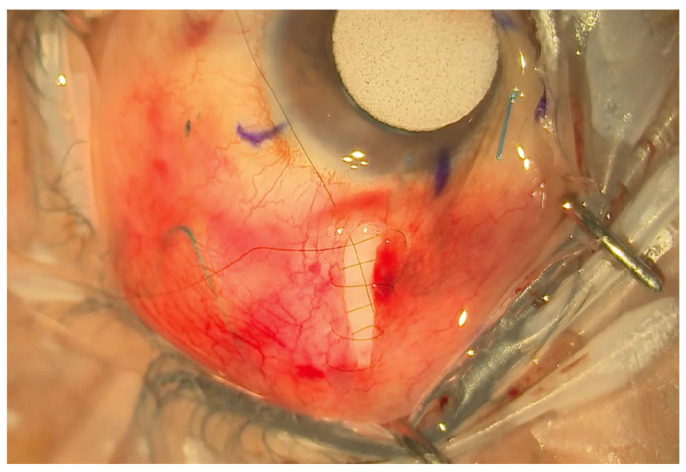
Small radial incision closed with a shoelace suture.

**Figure 4:**
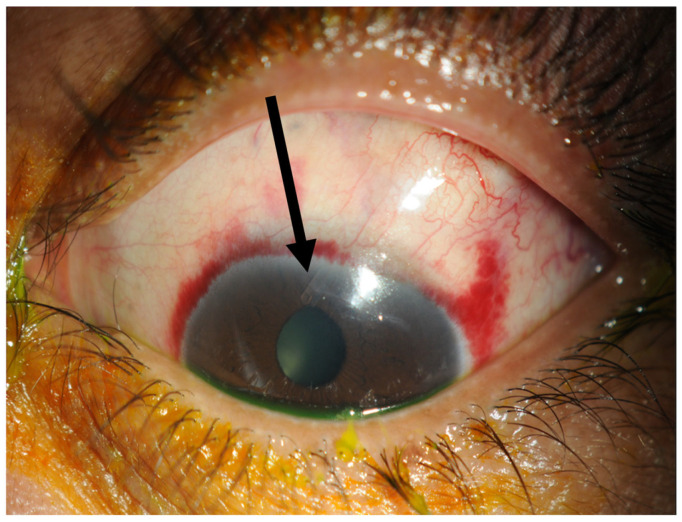
One week post-operatively. The tube marked with a black arrow is situated in the anterior chamber and the conjunctiva has already healed within one week.

A double layer of Tutoplast allograft tissue (Innovative Ophthalmic Products, Costa Mesa, CA, USA) is glued with Tisseel fibrin sealant (Baxter AG, Vienna, Austria). The 4/0 prolene stent suture end is tucked in the inferior fornix under the conjunctiva to facilitate ease of removal later in the outpatient setting. The end of the 4/0 prolene can be cauterised into a small bulb so it rests better under the conjunctiva and does not protrude through it. The smaller radial peritomy is then closed with Tisseel fibrin sealant glue and sutured with a 10/0 nylon suture. (Figure 3). To complete the surgery, a subconjunctival steroid and antibiotic are administered as well as an orbital floor 40 mg injection of methylprednisolone. See Figure 4 for a one-week post-operative outcome.

## 2. Methods

A retrospective review was conducted of 12 patients (13 eyes) in whom the eyePlate-300 implant was implanted using the MITS technique. All surgery was performed at our centre between December 2020 and September 2022.

During the pandemic, our centre looked at novel methods of glaucoma drainage device implantation. In March 2020, the centre started using the new eyePlate-300 tube—this was studied by our centre with a promising one-year pilot study [6]. From this tube’s unique design and plasticity, we then developed a new minimally invasive technique, starting in December 2020.

Ahmed et al. [6] published non-consecutive data between March 2020 and April 2021 of eyePlate-300 patients from our centre (note that none of these patients were included in this MITS study). During the overlap period of December 2020 and April 2021, when the new technique was being introduced, the senior surgeon continued to train surgeons on the implantation of the eyePlate-300 with the traditional technique whilst using the new technique on operations performed by the senior surgeon. From December 2020 onwards until the present, the senior surgeon operated on all patients receiving the eyePlate-300 tube with the new technique of minimally invasive tube surgery. 

The study was approved by the Imperial College New Interventional Procedures Committee, registered by the Imperial College NHS Trust as an audit, and adhered to the principles of the Declaration of Helsinki. All the enrolled patients were over the age of 18 years and gave written consent. The study followed the Declaration of Helsinki and its later amendments. The Imperial College New Devices Committee accepted MITS as a new technique.

Patients were offered GDD surgery if they had moderate-to-advanced glaucoma with uncontrolled intraocular pressure despite maximal tolerated topical therapy. Ten of the patients (77%) received general anaesthesia, and three patients (23%) received sub-tenons anaesthesia with lidocaine 2% and bupivacaine 0.5%. The same operating technique was employed in all procedures by the same surgeon (FA). All participants were monitored for a period of at least twelve months.

All patients received an orbital floor steroid depot consisting of 40 mg/mL methylprednisolone and a subconjunctival injection of cefuroxime/dexamethasone (3.3 mg/mL). Post-operatively, all patients were prescribed dexamethasone 0.1% preservative-free eye drops every two hours and chloramphenicol 0.5% preservative-free eye drops four times a day for a minimum of two weeks following the surgery.

The eyePlate-300 was approved for use by the Imperial College New Devices Committee. Our study adhered to the ‘World Glaucoma Association (WGA) Guidelines’ [7] on the design and reporting of glaucoma surgical trials to define the criterion for success. Failure was defined as an IOP out of the target range (5–20 mmHg inclusive), the need for further glaucoma surgeries in this eye to control the IOP, the need to remove the implant, or vision worsening to no perception of light (NPL) due to any reason.

Furthermore, we evaluated success rates in achieving an IOP range of 5 mmHg to 18 mmHg and 5 mmHg to 15 mmHg. Complete success was defined as reaching the IOP range without eye drops and qualified success with eye drops. Secondary outcomes were visual acuity, medication usage at 12 months, complication rates, and the need for further surgery.

Pre- and post-operative data were taken at intervals of 1 day, 1 week, 4 weeks, 3 months, 6 months, and 12 months following the surgery. At each visit, the following parameters were recorded: visual acuity, intraocular pressure (IOP) measured by a Goldmann applanation tonometry, and the number of topical agents used. Statistical analysis and graphs were produced using GraphPad Prism version 10. Descriptive statistics were described as the mean ± standard deviation for continuous variables and as a percentage for categorical variables. Statistical analysis was conducted to compare pre-operative and post-operative data using a one-way *t*-test. Results were considered statistically significant if the *p*-value was less than 0.01.

## 3. Results

### 3.1. Baseline Characteristics

Data for the thirteen eyes are outline below in Table 1, which shows information on the maximum intraocular pressure and pressure at timepoints week 1, week 4, month 3, and month 12. Table 1 also shows the pre- and post-operative drop use by the patients.

Baseline characteristics are summarised in Table 2. There were 12 patients and 13 eyes included in the study, with a mean age of 57.16 years. There was a 42:58 female-to-male ratio. We had an ethnically diverse population, with only three Caucasian patients (23%). Four (30%) patients had primary open-angle glaucoma (POAG), one patient (8%) had neovascular glaucoma, one patient (8%) had chronic angle-closure glaucoma, and two (25%) had uveitic glaucoma. The average number of ocular surgeries the patients had prior to undergoing the MITS technique was 1.42. This subjected subset was surgically complex, with some patients having undergone previous trabeculectomies, GDDs, cyclophotocoagulation, micro-invasive glaucoma surgery, vitreoretinal surgery, and corneal surgery.

### 3.2. Primary Outcome: Complete Success vs. Qualified Success

Seven eyes (54%) of our patients achieved complete success—with a pressure of less than 21 mmHg and completely off medication.

**Figure 5:**
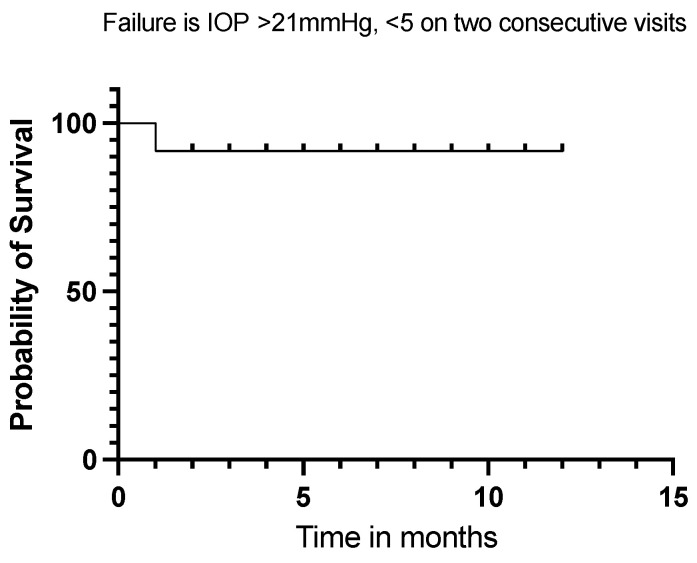
Kaplan–Meier Survival Analysis Curve showing a 92% qualified success when IOP is defined between 5 mmHg and 21 mmHg at 12 months.

**Figure 6:**
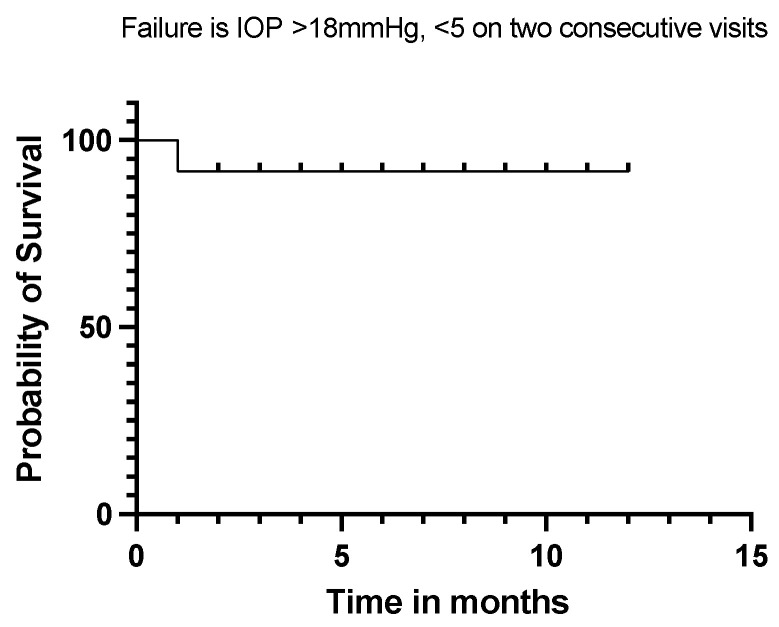
Kaplan–Meier Survival Analysis Curve showing a 92% qualified success when IOP is defined between 5 mmHg and 18 mmHg.

**Figure 7:**
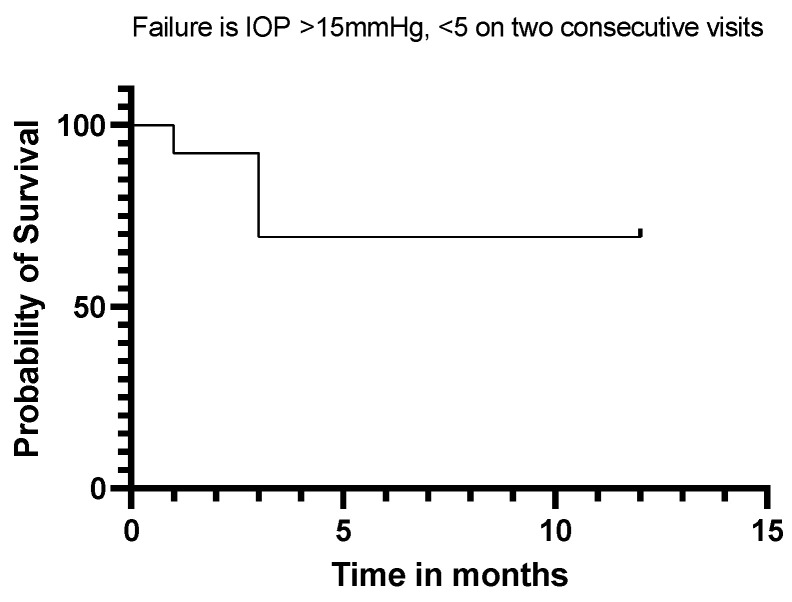
Kaplan–Meier Survival Analysis Curve showing a 69% qualified success when IOP is defined as 5 mmHg–15 mmHg.

Twelve (92%) of our eyes achieved qualified success when using IOP < 21 mmHg and IOP < 18 mmHg as the definition (see Figure 5 and Figure 6). Qualified success dropped to 69% when the IOP was defined as less than 15 mmHg (Figure 7).

**Figure 8:**
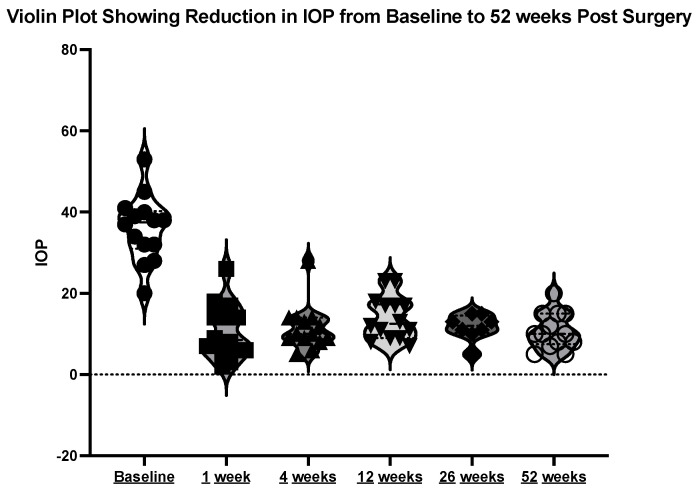
Violin plot showing reduction in IOP at the above timepoints.

The mean initial intraocular pressure (IOP) was 35.69 mmHg (±8.41), with a range between 20 and 53 mmHg. At week one post-operation, the mean IOP was 9.15 mmHg (±5.71). At week four post-operation, the mean IOP was 11.31 mmHg (±5.74). At month three post-operation, the mean IOP was 13.69 mmHg (±5.42). At month 12 post-operation, the mean IOP was 11.08 mmHg (±4.31). See the violin plot in Figure 8.

The mean difference in the IOP from pre-operation to 12 months post-operation was minus 24.62 mmHg (±2.863) *p* < 0.001.

### 3.3. Medication Usage

The mean pre-operative drops taken per patient were 3.462, with a range between three and four drops. There were six patients on maximal medical therapy (four drops), and the other seven patients were taking three drops.

**Figure 9:**
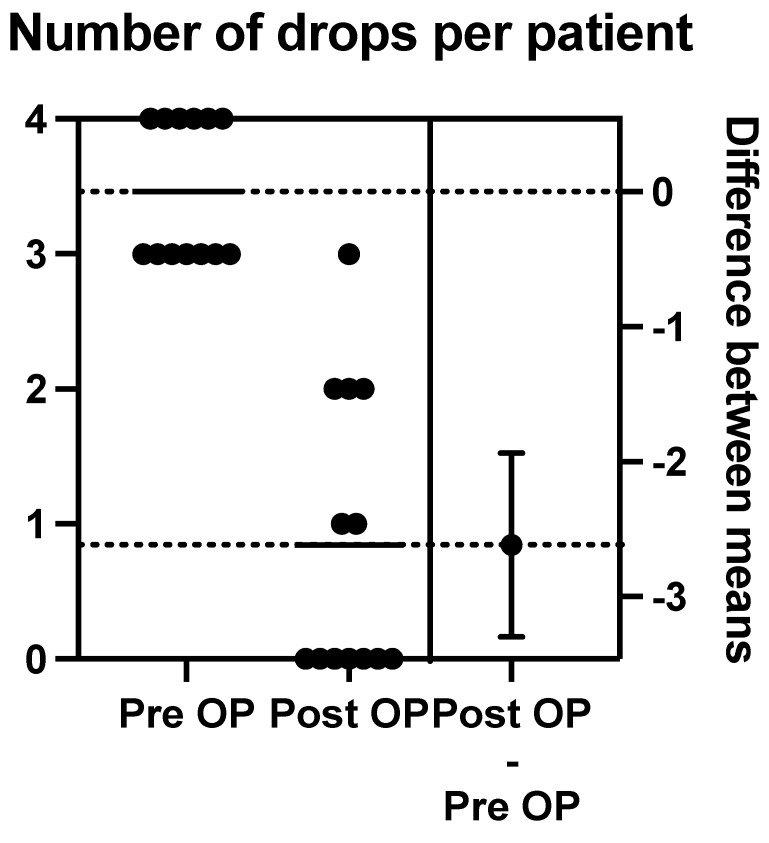
The above schematic outlines the change in pre-operative to post-operative number of drops per patient.

Post-operation at 12 months, we calculated the mean number of post-operative drops to be 0.85 (range of 0–3). Nine patients were on either no drops or one drop at 12 months post-operation (see Figure 9).

### 3.4. Visual Acuity

The pre-operative mean visual acuity, as recorded in LogMAR, was 1.354, with a range of 0.0–2.4 LogMAR. The post-operative mean visual acuity was 0.90 LogMAR. There was, therefore, no statistical difference in visual acuity at 12 months post-operation.

### 3.5. Complications

There were no intraoperative or post-operative complications to report in any of the 13 eyes operated on. None of our patients required further incisional surgery during the twelve-month follow-up period.

## 4. Discussion

Our study demonstrates a new technique for the implantation of a GDD using the minimally invasive tube procedure (MITS), with encouraging 12-month post-operation results. Our data on the complete success of the MITS technique using the WGA criteria show promising results at levels of 15 mmHg, 18 mmHg, and 21 mmHg thresholds. One hundred per cent of patients achieved IOPs between 5 mmHg and 21 mmHg, and 70% achieved between 5 and 15 mmHg at 12 months.

While the eyePlate-300 is similar to previous GDDs, it has a few notable exceptions, including its thin profile, which helps make the plate more flexible and allows its implantation via our MITS technique. This unique flexible characteristic allows the GDD to be folded in half “Taco style”, which allows it to be implanted through a relatively small keyhole conjunctival pocket incision, unlike traditional GDDs that require larger peritomies.

It is thought that the glaucoma drainage device’s success at reducing intraocular pressure is related to its plate areas and the fibrous capsule thickness that overlies the plate. A larger plate area and a thinner fibrous capsule is associated with lower long-term intraocular pressure [8,9]. The eyePlate’s plate has an almost square shape that allows it to fit between the recti muscles whilst still maintaining a large surface area (300 mm^2^). This makes the plate of the eyePlate-300 GDD only second behind the Baerveldt 350 in terms of the plate surface areas available in the market.

Tall bleb heights are associated with thick fibrous walls and higher IOPs, whereas flatter blebs are associated with thinner bleb walls and lower intraocular pressures. In the Ahmed versus Baerveldt study, the long-term lower intraocular pressure in the Baerveldt GDD was partly explained by the lower bleb height associated with it [10,11].

The eyePlate-300 also has two properties that encourage a lower bleb height; firstly, it has a thinner plate than the Baerveldt GDD, and additionally, the plate is more flexible, so when it is sutured to the sclera, it sits more snugly against the sclera, resulting in a very flat profile. This low-plate profile may contribute to favourable post-operative IOP outcomes at 12 months, even in a challenging population.

The MITS technique means the limbal architecture is unaffected, thus improving post-operative cosmesis and possibly comfort due to a smaller conjunctival pocket. Given that the plate fits between the two recti muscles due to its narrow profile, there is also, theoretically, a reduced incidence of post-operative diplopia. It should be highlighted that the MITS conjunctival pocket opening must be radial and not circumferential to minimise the risk of a leak post-operatively due to the radial tissue tension. Indeed, the radial wound used in MITS is easier to close, with the need for fewer sutures.

Our patient cohort was an ethnically diverse cohort, in keeping with the London population, with only 36.8% being White Caucasian. Despite this, twelve out of the thirteen eyes achieved a statistically significant reduction in IOP, with only one of our patients having a pressure that remained the same after twelve months (20 mmHg), but they were on fewer glaucoma medications. Our results show a good mean reduction from the baseline at each time point identified over the 12 months.

Three out of our cohort underwent the procedure under local anaesthesia, with the remainder undertaken under general anaesthesia, which has been the preferred choice of surgeons performing GDD surgery. The COVID-19 era has redefined many areas of ophthalmology, and anaesthetic choice is one, with our patients tolerating a local anaesthetic well. Furthermore, the minimally invasive technique should be tolerated even more than traditional tube surgery methods due to various factors: not having to interfere with the recti muscles, potentially reduced time for surgery and a reduced need for suturing intraoperatively.

Potential complications arising from the MITS technique may involve trapping the plate in a recti muscle due to a lack of visualisation when the plate unfolds through the small peritomy. This risk is mitigated by the authors’ recommendation of hooking the muscle to identify insertions, in addition to the long rectangular shape of the eyePlate-300 that naturally sits between the superior and lateral rectus, with a superotemporal approach. Another complication could be conjunctival trauma when inserting the folded plate. This is mitigated by creating a continuous linear incision with no microtears; this will ensure the conjunctival pocket that is created is large. If the conjunctiva does tear out, then one can adapt to the more traditional method of inserting the GDD using a larger peritomy.

The following limitations and recommendations should be considered in our study. We had a relatively small non-randomised sample size. The small sample size may restrict generalisability, and therefore, we would recommend further research into the MITS technique with a higher sample size. There was a short duration of follow-up at 12 months, which is relatively brief for determining the long-term efficacy and safety of a new surgical method; the authors recommend that a longer duration should be studied in future research work. All cases were selected from a single site, and surgery was performed by a single surgeon, which could have potentially introduced a bias. In future research, we would recommend a multicentre study with multiple surgeons. Given that this is a new technique, we would recommend a further study comparing MITS to traditional GDD implantation. A final limitation to discuss would be statistical analysis; future work could involve the use of a more sophisticated statistical analysis, including multivariate analyses to account for any confounding variables.

## 5. Conclusions

We demonstrated a novel and reproducible technique of implanting a glaucoma drainage device through a small conjunctival incision. This technique avoids the need for a large limbal peritomy, causing minimal conjunctival trauma and effectively lowering intraocular pressure. We would recommend further research into the MITS technique, looking at a larger patient profile with longer follow-up intervals in a multicentre study.

## Figures and Tables

**Table 1:** Showing patient data for the pre-operative intraocular pressure, pressure at weeks 1, 4, 12 and 52 and pre- and post-operative drop use.

Patient	Pre-Op Maximum IOP	Week 1 Post-Op	Week 4 Post-Op	Month 3 Post-Op	Month 12 Post-Op	Drops Pre-Op Total	Drops 12 Months Post-Op Total
1	53	14	13	9	12	3	0
2	38	14	14	11	10	4	0
3	27	17	6	18	15	3	0
4	39	6	14	9	15	4	0
5	38	7	9	8	8	3	0
6	34	8	10	13	10	4	0
7	32	26	28	23	10	3	2
8	32	18	9	12	5	3	2
9	45	9	5	23	8	3	2
10	41	14	10	11	11	4	1
11	20	2	9	17	20	4	1
12	37	3	12	17	15	3	3
13	28	6	8	7	5	4	0

**Table 2:** Baseline characteristics of the study group.

Number of patients (number of eyes)	12 (13)
Mean age	57.16 years
Gender (%)	Female: 5 (42%)
	Male: 7 (58%)
Mean Number of Previous Surgeries: Ethnicity (%)	1.42
	Caucasian 3 (23%) Afro-Caribbean 3 (23%)
	Asian 2 (15%)
	Indian Subcontinent 5 (39%)
Laterality (%)	Left: 54%
	Right: 46%
Visual acuity (LogMAR)	Mean 1.35 (±1.007) range 0.0 to 2.4 (equivalent to Snellen 6/6—HM)
Medications Use:	3.462 (±0.5175)
Diagnosis (%)	Secondary Glaucoma (8%) Chronic Angle Closure Glaucoma (8%) Primary Open Angle Glaucoma (30%) Neovascular Glaucoma (8%) Uveitic Glaucoma (46%)
Previous Surgeries	Vitreoretinal *n* = 3 Corneal *n* =1 Cyclophotocoagulation *n* = 2 Micropulse Laser Trabeculoplasty *n* = 1 Phacoemulsification *n* = 2 MIGS *n* = 2 Microshunt *n* = 2 Glaucoma Drainage Device *n* = 2 Peripheral Iridotomy *n* = 1 Trabeculectomy *n* = 2

## Data Availability

Dataset on request.
